# Radiation Dose Reduction in Cancer Imaging with New-Model CT Scanners: A Quality of Care Evaluation

**DOI:** 10.3390/cancers17111815

**Published:** 2025-05-29

**Authors:** Stefania Rizzo, Luca Bellesi, Ebticem Ben Khalifa, Stefano Presilla, Andrea D’Ermo, Francesco Magoga, Matteo Merli, Ermidio Rezzonico, Oriana D’Ecclesiis, Filippo Del Grande

**Affiliations:** 1 Clinic of Radiology, Imaging Institute of Southern Switzerland (IIMSI), Ente Ospedaliero Cantonale (EOC), 6900 Lugano, Switzerlandfrancesco.magoga@eoc.ch (F.M.);; 2 Faculty of Biomedical Sciences, Università Della Svizzera Italiana (USI), Via G. Buffi 13, 6904 Lugano, Switzerland; 3 Service of Medical Physics, Imaging Institute of Southern Switzerland (IIMSI), Ente Ospedaliero Cantonale (EOC), 6500 Bellinzona, Switzerland; luca.bellesi@eoc.ch (L.B.);; 4 Service of Process Organization and Information, Support Area, Ente Ospedaliero Cantonale (EOC), Via Lugano 4D, 6500 Bellinzona, Switzerland; 5 Independent Researcher, 00100 Rome, Italy

**Keywords:** computed tomography, replacement, radiation dose, computed tomography dose index, dose length product, objective image quality

## Abstract

Background/Objectives: The primary aim of this study was to evaluate whether the replacement of roughly one-decade-old computed tomography (CT) scanners with new-model CT scanners were associated with an additional reduction in the radiation dose delivered to oncological patients, in a radiological setting where the optimization of protocols had already reached very low radiation doses. An exploratory secondary objective was to evaluate the potential differences in the objective image quality between the CT scans obtained before and after the installation of the new-generation CT scanners. Methods: Chest and abdominal CT examinations conducted for oncologic purposes were retrospectively selected from two time periods—prior to scanner replacement (2022) and following an upgrade (2024)—after five CT systems in our radiology department were replaced. We extracted and compared the CT dose index (CTDI) and dose length product (DLP) for each CT phase. For the objective image quality evaluation, we calculated the signal-to-noise ratio (SNR) and the contrast-to-noise ratio (CNR) at the center of the liver and the aorta. An appropriate statistical analysis was performed and a *p*-value < 0.05 was considered significant. Results: We included 14,601 CT acquisitions, of which 9013 (61.7%) were performed before and 5588 (38.3%) after the replacement of the CT scanners. There were significantly lower values for the CTDI and DLP with the new CT scanners compared to the old ones. The CTDI with the new CT scanners was significantly lower in all phases (*p*-value = 0.002 for unenhanced phase, and *p* < 0.001 for arterial, portal venous, and delayed phases). The DLP using the new CT scanners was significantly lower in the arterial, portal venous, and delayed phases (*p* < 0.001), and it was not significantly different in the unenhanced phase (*p* = 0.36). There was no significant difference in the SNR at the liver level (*p* = 0.72) or at the aorta level (*p* = 0.51). There was no significant difference in the CNR at the liver level (*p* = 0.24), whereas the CNR was higher with the new CT scanners at the aorta level (*p* = 0.03). Conclusions: The transition to new-model CT scanners resulted in a significant reduction in the radiation dose delivered by chest and abdomen CT scans, without compromising the objective image quality.

## Simple Summary

This study aimed to assess whether replacing computed tomography (CT) scanners with new models reduced the radiation doses in oncological patients, even in a setting with optimized protocols. It also evaluated the impact on the objective image quality before and after scanner replacement. This study included 14,601 chest and abdomen CT scans. The results showed significant reductions in the radiation doses (CTDI and DLP) with the new-model CT scanners in all CT phases except the unenhanced phase. The image quality, as assessed by the signal-to-noise ratio (SNR) and contrast-to-noise ratio (CNR), showed no significant variation at the liver or aorta across the scanners; however, the CNR was elevated at the aortic level with the newer systems. These results indicate that upgrading CT scanners may allow for reduced radiation exposure without compromising the image quality.

## 1. Introduction

Since its inception in the 1970s, computed tomography (CT) has seen a consistent rise in use worldwide [1]. In the United States alone, the CT scan volume increased by about 20% between 2006 and 2016, while global figures showed nearly a twofold increase during the same period [2]. In the US, by 2016, there were an estimated 230 CT exams per 1000 people, with the largest categories being CT exams of the abdomen and pelvis (20.1 million procedures), brain (15.3 million), and chest (12.7 million) [2,3]. Several factors have contributed to the growing use of CT in medical diagnostics. In fact, CT scanners have become indispensable in both emergency and routine settings due to their widespread availability in medical facilities, from major hospitals to smaller regional centers. They offer 24/7 accessibility and are user-friendly for both healthcare providers and patients, thanks to the speed and efficiency of CT examinations. Furthermore, advancements in CT technology have significantly extended the axial coverage in a single rotation, enabling optimal three-dimensional reconstructions and the simultaneous evaluation of multiple body regions [4,5,6,7].

With the growing utilization of CT imaging, concerns have emerged regarding radiation exposure, especially among patients who undergo repeated scans over time. Guidelines have been established emphasizing the need for justification, dose optimization, and adherence to recommended dose limits [8,9].

It is, therefore, no surprise that radiation dose reduction in CT scanning has become a critical focus for both equipment manufacturers and healthcare providers. In this regard, alongside the increase in the number of CT exams performed and the number of scans per person, the total collective effective dose in the U.S. population has decreased since 2006, from 885,000 person-sieverts in 2006 to 717,000 person-sieverts in 2016 [3]. The main reasons for this decline include heightened awareness of radiation doses, increased education, dose optimization by medical physicists, changes in practices, and newer technologies.

Our department, like many others, has made significant efforts to reduce the radiation dose associated with CT scans [10], as recognized by our classification as five-star premium by the Eurosafe initiative of the European Society of Radiology in 2023. During the year 2024, we replaced five over 10-year-old CT scanners with recent models. Our CT protocols had already been optimized [10], and when the new CT scanners were placed on site, we transferred our optimized protocols to the new scanners, with subtle adaptations related to the new technology.

Keeping in mind the concept that technological advances in CT scanners may contribute to further reductions in radiation doses [4,7,11], the main aim of this study was to determine whether the installation of the new CT scanners in our department led to a further reduction in the radiation dose for oncologic patients undergoing chest and abdominal CT imaging. As a secondary exploratory objective, we assessed whether the objective image quality differed between the examinations conducted before and after the scanner upgrade.

## 2. Materials and Methods

The present study was a quality care study, which did not fall under the Swiss law regulating human research; therefore, it did not require specific approval of the local ethical committee (Req-2025-00392, Rif CE TI 4817). All the data were anonymized and grouped for statistical analysis.

CT exams selection: Chest and abdominal CT scans performed for oncologic indications were randomly selected using a business intelligence and visualization tool (Microsoft Power BI Desktop). The scans were acquired over a 12-month period (1 March 2023–29 February 2024) using five different CT scanners: four SOMATOM Definition Edge systems (Siemens AG, Erlangen, Germany; introduced in 2011) and one Brilliance iCT scanner (Philips Healthcare, Eindhoven, The Netherlands; introduced in 2007). CT scans obtained after the replacement of the abovementioned CT scanners (including the purchase of an additional one) with 6 brand new CT scanners (5 SOMATOM X.ceed, Siemens Healthineers AG, Erlangen, Germany, first introduced in 2021; 1 SOMATOM X.cite, Siemens Healthineers AG, Erlangen, Germany, first introduced in 2019) were also extracted for a 10-month period (1 March 2024–31 December 2024).

Examinations were excluded based on the following criteria: scans present in the local Picture Archiving and Communication System (PACS) but acquired at external institutions; presence of metallic prostheses due to their potential impact on radiation dose [12]; inclusion of CT phases involving the head when performed in conjunction with chest and abdominal imaging; and series limited to localizers or a small number of images, such as bolus-tracking sequences.

Dose report extraction: Radiation output for each CT phase acquisition (unenhanced, arterial, portal venous, and delayed phases) was obtained using a commercially available dose management platform (Radimetrics, Bayer HealthCare, Leverkusen, Germany) implemented at our institution. To minimize bias related to protocol variability in the number of phases, radiation metrics were analyzed per acquisition rather than per patient. The extracted parameters included the CT dose index (CTDI, in mGy) and the dose length product (DLP, in mGy x cm).

Objective image quality evaluation: a group of 120 CT scans was randomly selected from the CT scans performed before (n = 60) and after (n = 60) the replacement of the CT scanners.

The images were anonymized and categorized into two groups for objective image quality analysis. On a single axial slice at the level of the first lumbar vertebra (L1), three circular regions of interest (ROIs) were manually placed to assess tissue density in the following structures: the abdominal aorta (covering at least two-thirds of the lumen without touching its borders), the right hepatic lobe (targeting a homogeneous parenchymal area), and the right paraspinal muscles [10]. For each ROI, the mean and standard deviation of attenuation values (measured in Hounsfield units, HU) were recorded. These values were then used to compute signal-to-noise ratio (SNR) and contrast-to-noise ratio (CNR) for both the liver and the aorta using the following formulas [13]:

SNR_liver = HU_liver/SD_liver

SNR_aorta = HU_aorta/SD_aorta

CNR_liver = (HU_liver − HU_muscle)/√(SD_liver^2^ + SD_muscle^2^)

CNR_aorta = (HU_aorta − HU_muscle)/√(SD_aorta^2^ + SD_muscle^2^)

### Statistical Analysis

Kolmogorov–Smirnov test, Shapiro test, and QQplot were used to test the normality of CTDI, DLP, SNR and CNR distributions. Since the dose descriptors were not found to be normally distributed, the nonparametric Wilcoxon–Mann–Whitney test was used to compare the distribution of both the dose descriptors and objective image quality (in terms of SNR and CNR) between the old and new CT scanners. Data were reported as median values with interquartile ranges (IQRs). A two-tailed *p*-value of less than 0.05 was considered indicative of statistical significance. All analyses were conducted using R software (version 4.3.2).

## 3. Results

In our department, we perform about 4000 thorax and abdomen CT scans for oncologic indications yearly. In this study, we included 14,601 CT phases, of which 9013 (61.7%) were from before and 5588 (38.3%) were from after the replacement of the CT scanners. The detailed numbers for the CT phases (unenhanced arterial, venous, and delayed phases) included are summarized in Table 1.

**Figure 1:**
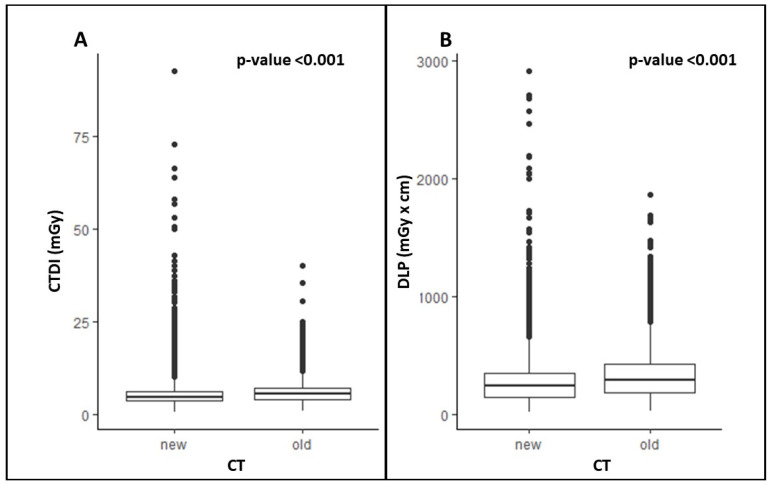
Differences in CTDI (**A**) and DLP (**B**) for all CT phases from the old and new CT scanners.

The CTDI and DLP are standardized metrics used to evaluate the radiation dose a patient receives during a CT scan. The CTDI measures the dose per slice, while the DLP is related to the total radiation dose based on the scan length. In this study, we found an overall significant difference in the CTDI and DLP values between the two groups of CT scans (old = before and new = after the replacement of the scanners), with significantly lower values for the new CT scanners (Figure 1) compared to the old ones.

As shown in Table 2, the CTDI was significantly lower for the new CT scanners compared to the old ones. This was true across all acquisition phases (unenhanced, arterial, venous, and delayed), with the *p*-values indicating statistically significant reductions with the new scanners (ranging from *p* < 0.001 to 0.002). Similarly, the DLP was also significantly lower with the new scanners in all phases except for the unenhanced phase, which showed no significant difference (*p* = 0.36). The *p*-values for the other phases (arterial, venous, and delayed) were all <0.001, indicating substantial reductions in the total radiation dose with the new scanners. These results suggest that the new CT scanners are significantly more efficient in terms of the radiation dose, offering lower radiation exposure to patients while maintaining the necessary imaging quality.

### 3.1. CTDI

**Figure 2:**
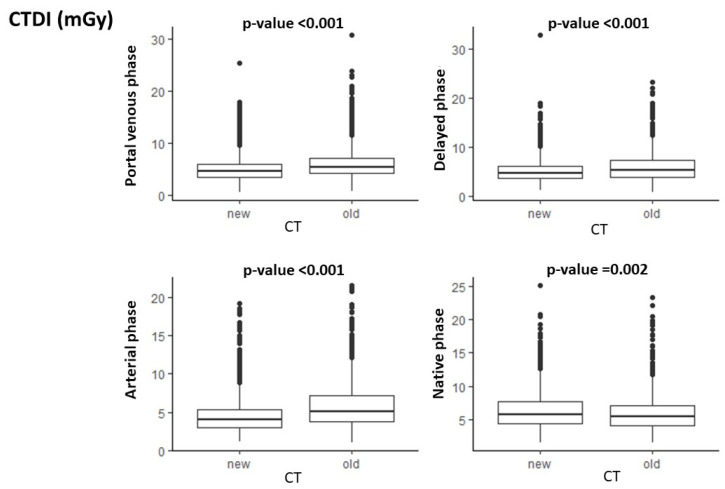
Differences in CTDI for each phase of acquisition with old and new CT scanners.

As shown in Figure 2, there was a significant difference for the CTDI in all CT phases acquired with the old CT scanners compared with the new ones, with lower values for the CT exams acquired with the new scanners (*p*-value = 0.002 for unenhanced phase, and *p* < 0.001 for arterial, portal venous, and delayed phases).

### 3.2. DLP

**Figure 3:**
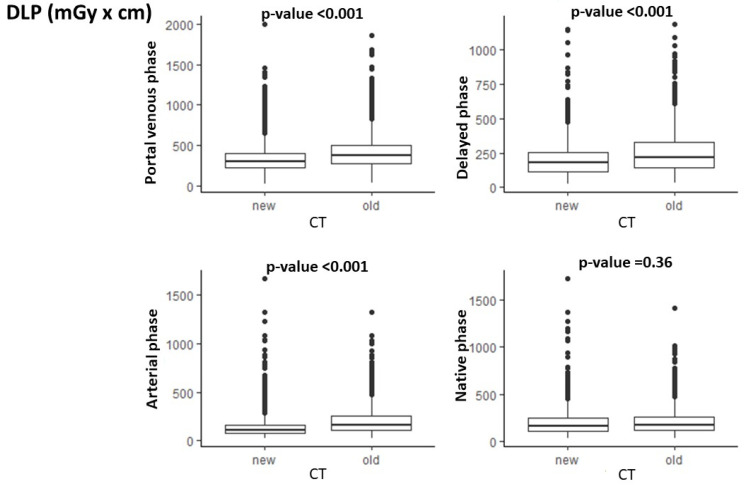
Differences in DLP for each phase of acquisition with old and new CT scanners.

As shown in Figure 3, there was a significant difference for the DLP in the CT phases acquired with the old scanners compared with the new ones, with lower DLP values for the new scanners in all the acquisition phases (*p* < 0.001 in the arterial, portal venous, and delayed phases), with the sole exception of the unenhanced phase (*p* = 0.36).

### 3.3. SNR

The SNR is an objective measure of image quality, where higher values indicate clearer images (less noise). The objective image quality assessment showed no significant difference in the SNR in the CT exams acquired with the old scanners compared to the new ones at the liver level (*p* = 0.72) or at the aorta level (*p* = 0.51) (Table 3). This suggests that despite the lower radiation dose with the new scanners, the image quality is maintained at a comparable level.

### 3.4. CNR

The **CNR** measures the contrast in an image relative to the noise level, indicating how well different structures can be distinguished on a scan. In this study, we found no significant difference for the CNR in the CT exams acquired with the old scanners compared to the new ones at the liver level (*p* = 0.24). However, as shown in Table 4, the CNR at the aorta level was higher for the new scanners (*p* = 0.03), suggesting that the new CT scanners provide better contrast resolution at the aorta level, which might be clinically relevant for identifying structures or abnormalities in that area.

## 4. Discussion

### 4.1. Radiation Dose

#### 4.1.1. Radiation Dose Reduction

At a center where the CT protocols were already largely optimized to keep the radiation doses well below the diagnostic reference levels recommended for clinical imaging by the ICRP [9], we demonstrated that the upgrade to newer-model CT scanners was associated with a further significant reduction in both the CTDI and DLP. This dose reduction was consistent across the contrast-enhanced CT phases, suggesting that newer-model CT scanners are more efficient at visualizing iodine contrast.

Additionally, the evaluation of the objective image quality revealed no significant difference in the SNR between the scans acquired before and after the CT scanner upgrade. Notably, the CNR at the aortic level was slightly improved with the newer scanners.

It is worth noting that CT and nuclear medicine procedures account for approximately 75.4% of the total cumulative effective radiation dose, with 81.8% of this exposure occurring in outpatient settings [14]. Given this high number of CT exams performed, which is progressively increasing [2,9], there has been increasing attention to the radiation dose delivered by these exams [15,16], with a recent paper raising a warning about the cancer risk related to CT exams [17]. Indeed, it has been demonstrated that clinical CT scans can vary a lot in their dose and image quality, with broad implications for diagnostic utility and radiation burden. For instance, Smith et al. evaluated 87.629 CT scans, and demonstrated that across different facilities, the image quality and radiation doses were significantly different, with the scanner model, slice thickness, reconstructed field-of-view, mAs, kVp, patient size, and centering being the most influential factors [18].

#### 4.1.2. CT Technology Updates Contributing to Radiation Dose Reduction

The goal of all CT vendors is to develop new technologies that can increase the image quality for diagnostic purposes [5,19,20], while keeping the radiation dose constant or decreasing it. The most relevant technologies used for dose reduction in state-of-the-art CT scanners are automatic exposure control, which modulates the tube current based on the patient’s attenuation in the longitudinal direction and the image plane [21], or as an organ-based modulation [5,22]; iterative reconstructions, which can significantly reduce the image noise compared to the filtered back projection while maintaining image quality and detail visualization [5,23,24,25,26]; and Z-collimation, which prevents the dose coming from the part of the wide multislice fan beam at the scan beginning and end from contributing to the final images [27,28].

A major and more recent contribution to dose reduction has been achieved by shaping the X-ray spectrum more toward an ideal spectrum, which is achieved by adding a tin filter for the stronger filtration of the low-energy components of X-ray beams [29,30,31].

Consistently with the abovementioned technological advancements, Qurashi et al. demonstrated that iterative reconstructions can compensate for the noise increase in low-radiation-dose abdominal CT scans of larger-sized patients, and discussed the general impact of obesity on abdominal organ doses and image quality in CT scans [32]. Another study demonstrated that the simultaneous use of automatic tube current modulation and automatic tube voltage selection for abdominal and chest CT examinations enabled a significant radiation dose reduction compared with the use of automatic tube current modulation alone [33,34]. Rogalla et al. demonstrated that contiguous helical scanning of the chest/abdomen/pelvis with variable target noise levels resulted in an approximately 17% dose reduction, when compared to a single acquisition with only abdominal dose settings or two separate acquisitions of the chest and abdomen/pelvis [35], and Lee et al. demonstrated that the dose can be lowered by using an in-house device in patients undergoing CT scans without elevating the arms [36].

Our results build on these findings, and they extend the evidence base to a radiological setting where optimization efforts and protocols adjustments, according to a subspecialty organization, had already led to lower radiation doses [10].

In our study, both the older- and new-model CT scanners offer advanced technologies for reducing the radiation exposure, such as dose modulation and iterative reconstruction. The new-model CT scanners, SOMATOM X.ceed and SOMATOM X.cite, also feature tin filtration, further reducing the radiation dose patients are exposed to. These scanners also feature a FAST 3D camera, which utilizes artificial intelligence-based algorithms to capture a patient’s shape and position and facilitates more optimized patient positioning. This feature helps correct the positioning of a patient at the isocenter of a CT scanner, leading to a further reduction in the effective dose received from CT examinations [37,38]. Furthermore, these two recent-model CT systems allow for tube voltage adjustments in 10 kV increments between 70 kV and 150 kV, enabling more precise adaption to individual patients, optimizing the image quality and reducing the radiation exposure. Last but not least, SOMATOM X.ceed and SOMATOM X.cite contain automated support for protocol adjustment (myExam Companion), which helps optimize the tube voltage, current, and reconstruction settings, helping to achieve the lowest possible dose while maintaining diagnostic image quality. These features may have contributed to the lower radiation exposure observed with the newer-model CT scanners included in this study.

As a result, in our cohort, replacing the older CT scanners with newer models featuring current technology provided an added benefit in terms of a radiation dose reduction, as measured by the CTDI and DLP. This highlights the importance of regularly upgrading medical imaging equipment to benefit from the latest advancements in radiation safety. Specifically, the CTDI was significantly lower across all phases, while the DLP showed no significant difference between the two groups, except in the unenhanced phase. The CTDI decrease, while the DLP remained unchanged, may have been due to an increase in the scan length or automatic dose modulation adjustments compensating for the reduction. Nevertheless, it is important to note that the DLP in the unenhanced phase was not higher than the DLP obtained with the old scanners. In our opinion, the results of this study point out that the replacement of old CT scanners with new ones shows a positive effect on the radiation exposure of patients, with lower doses associated with the new scanners, and this is important, despite possible slight increases in radiation doses in single phases.

### 4.2. Objective Image Quality

Despite the reduction in the radiation dose, no significant difference was observed in the objective image quality (SNR and CNR at the liver level) between the CT scans from the older- and newer-model scanners. This finding suggests that modern CT scanners are able to maintain high diagnostic image quality while achieving lower radiation doses, which is crucial for patient safety. On the one hand, the lack of significant changes in the SNR supports the idea that technological advancements allow for a balance between dose reduction and image quality, while on the other, the improvement in the CNR at the aorta level suggests that the newer scanners may enhance the image quality of certain anatomical regions without an associated increase in the radiation dose.

Our results may have interesting consequences for oncologic imaging. Indeed, oncological patients often require repeated CT scans for treatment planning and monitoring of the disease and of the therapies’ effects [39,40,41,42,43]. The reduction in radiation exposure is related to a reduction in the cumulative effective dose and could potentially lead to a reduction in the risk of radiation-induced malignancies over time. This is particularly important in cancer patients who, if diagnosed at early stages or showing targetable mutations, could potentially be long-term survivors [44]. Additionally, an even improved CNR at the aorta level may enhance diagnostic confidence in certain clinical settings, providing an additional benefit in terms of diagnostic accuracy.

### 4.3. Limitations

While this study provides valuable insights into the potential benefits of replacing old CT scanners, there are some limitations that should be considered. First, this study was conducted at a single institution. However, the large sample size (number of CT phases = 14,601) and the inclusion of both chest and abdomen CT scans across multiple phases strengthens the generalizability of our results. Moreover, the inclusion of CT scans performed before and after the replacement of CT scanners at the same center minimized the confounding variables, as all patients were imaged using equivalent protocols, and a change in the CT technology was the only major intervention. Second, the assessment of image quality relied only on objective measures, without considering subjective evaluations by radiologists. Further studies incorporating subjective assessments by radiologists may provide additional insights into the clinical significance of these findings. Third, this study did not account for potential differences in patient demographics or tumor types that might have influenced the results. Nevertheless, this was a quality of care study, to assess whether the technology replacement led to a further reduction in the radiation dose, and was not meant to evaluate single or grouped patients according to their specific clinical conditions or the cancer type with which they were affected. Future studies could explore the impact of CT scanner replacement on specific patient populations, to assess whether dose reductions and image quality outcomes are associated with clinical conditions. Additionally, we did not assess the impact of newer technologies, such as photon-counting CT, on dose reduction. Unfortunately, our department is not yet equipped with this latest technology, but we do expect to see further improvements in dose reduction related to this new technology, given the reduced beam hardening and electronic noise, along with increased spatial resolution [45,46]. Lastly, we did not assess if there was a difference in the dose reduction according to different vendors, because all the new CT scanners were from the same vendor. However, we do believe that all CT vendors take care regarding the radiation dose, and they ameliorate the related-technology overtime in new scanner models; therefore, these results should be reproducible with CT scanners from different vendors.

## 5. Conclusions

The replacement of old CT scanners with new ones offering technological advancements is associated with a significant reduction in the radiation dose delivered to oncological patients, even in a setting where the protocols were already optimized to reduce radiation exposure, without compromising image quality. These findings support the continued adoption of new CT technologies to enhance patient safety and diagnostic performance in oncological imaging.

## Figures and Tables

**Table 1:** Number of CT phases included for old (years 2007 and 2011) and new (year 2024) CT scanners.

	CT Phases	
	Old CT Scanners N (%)	New CT Scanners N (%)
Unenhanced	1254 (64.7)	683 (35.3)
Arterial	1869 (63.8)	1059 (36.2)
Venous	5002 (60.6)	3254 (39.4)
Delayed	888 (60.0)	592 (40.0)

**Table 2:** CTDI and DLP values for the old and new CT scanners, overall and for each single phase of acquisition.

	Old CT Scanners Median (IQR)	New CT Scanners Median (IQR)	*p*-Value
CTDI			
Overall	5.40 (4.08–7.14)	4.63 (3.55–6.20)	<0.001
Phase			
Unenhanced	5.42 (4.07–7.13)	5.72 (4.34–7.67)	0.002
Arterial	5.13 (3.75–7.10)	4.01 (3.00–5.32)	<0.001
Venous	5.40 (4.17–7.09)	4.56 (3.57–5.97)	<0.001
Delayed	5.30 (3.95–7.34)	4.72 (3.65–6.21)	<0.001
DLP			
Overall	288.6 (180.6–423.0)	244.0 (148.0–352.0)	<0.001
Phase			
Unenhanced	177.6 (119.6–262.9)	168.0 (116.0–250.0)	0.36
Arterial	164.9 (107.1–256.0)	112.0 (77.8–163.0)	<0.001
Venous	370.2 (277.2–497.8)	297.0 (228.0–399.0)	<0.001
Delayed	215.7 (144.0–329.4)	177.5 (112.8–257.5)	<0.001

CTDI = computed tomography dose index; DLP = dose length product; IQR = interquartile range.

**Table 3:** Table showing median and interquartile range (IQR) of signal-to-noise ratio (SNR) at the level of liver and aorta.

	Median (IQR)	*p*-Value for Difference
Liver		0.72
Old CT scanners	8.59 (7.29–9.63)	
New CT scanners	8.47 (7.40–9.39)	
Aorta		0.51
Old CT scanners	10.9 (9.81–11.9)	
New CT scanners	10.5 (8.90–12.57)	

**Table 4:** Table showing median and interquartile range (IQR) of contrast-to-noise ratio (CNR) at the level of liver and aorta.

	Median (IQR)	*p*-Value for Difference
Liver		0.24
Old CT scanners	2.55 (2.22–3.19)	
New CT scanners	2.86 (2.28–3.42)	
Aorta		0.03
Old CT scanners	4.67 (3.91–5.52)	
New CT scanners	5.23 (4.17–6.38)	

## Data Availability

Grouped anonymized data may be available upon reasonable request.

## Abbreviations

The following abbreviations are used in this manuscript (in alphabetical order):

CNR	Contrast-to-Noise Ratio
CT	Computed Tomography
CTDI	Computed Tomography Dose Index
DLP	Dose Length Product
IQR	Interquartile Range
PACS	Picture Archive and Communication System
SNR	Signal-to-Noise Ratio
